# Extrinsic and intrinsic regulation of axon regeneration at a crossroads

**DOI:** 10.3389/fnmol.2015.00027

**Published:** 2015-06-16

**Authors:** Andrew Kaplan, Stephan Ong Tone, Alyson E. Fournier

**Affiliations:** Department of Neurology/Neurosurgery, Montréal Neurological Institute, McGill UniversityMontréal, QC, Canada

**Keywords:** spinal cord injury, CNS regeneration, PNS, conditioning lesion, RhoA, cAMP

## Abstract

Repair of the injured spinal cord is a major challenge in medicine. The limited intrinsic regenerative response mounted by adult central nervous system (CNS) neurons is further hampered by astrogliosis, myelin debris and scar tissue that characterize the damaged CNS. Improved axon regeneration and recovery can be elicited by targeting extrinsic factors as well as by boosting neuron-intrinsic growth regulators. Our knowledge of the molecular basis of intrinsic and extrinsic regulators of regeneration has expanded rapidly, resulting in promising new targets to promote repair. Intriguingly certain neuron-intrinsic growth regulators are emerging as promising targets to both stimulate growth and relieve extrinsic inhibition of regeneration. This crossroads between the intrinsic and extrinsic aspects of spinal cord injury is a promising target for effective therapies for this unmet need.

## Introduction

**Figure 1:**
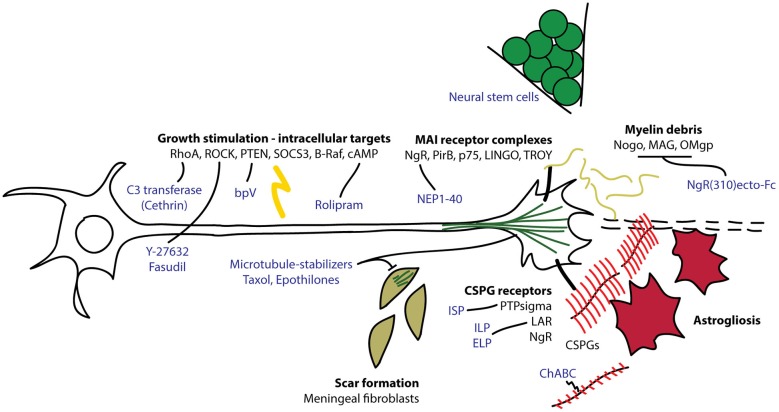
**Overview of intrinsic and extrinsic molecules involved in axon regeneration after CNS injury**. Myelin debris from damaged oligodendrocytes and chondroitin sulfate proteoglycans (CSPGs) produced by reactive astrocytes interact with receptors on the growth cones to interfere with axon extension. Intracellular signaling molecules regulate the ability of the neuron to regenerate its damaged axon through the lesion environment. C3 transferase (Cethrin) inhibits RhoA to promote axon extension and cell survival. Fasudil and Y-27632 inhibit Rho kinase (ROCK) to promote axon extension. Bisperoxovanadium (bpV) inhibits phosphatase and tensin homolog (PTEN) to promote neuroprotection. Rolipram inhibits phosphodiesterase 4 (PDE4) to stabilize cAMP and promote axon extension. Microtubule stabilizers, taxol and epothilones, reduce fibrotic scarring and promote axon extension. Chondroitinase ABC (ChABC) digests CSPG glycosaminoglycan (GAG) chains (in red), relieving CSPG-dependent growth inhibition. Intracellular sigma peptide (ISP) inhibits protein tyrosine phosphatase sigma (PTPsigma) and intracellular/extracellular leukocyte common antigen related phosphatase (LAR) peptides (ILP/ELP) inhibit leukocyte antigen-related receptor (LAR) to neutralize the inhibitory effect of CSPGs and promote axon sprouting. NgR(310)ecto-Fc competes for binding to Nogo, myelin-associated glycoprotein (MAG) and Oligodendrocyte myelin glycoprotein (OMgp) to relieve myelin-dependent growth inhibition. Nogo extracellular peptide 1–40 (NEP1–40) competes for NgR binding to neutralize inhibitory signaling through NgR.

The adult mammalian central nervous system (CNS) has a poor ability to regenerate and restore function after injury. The pioneering work of Aguayo and colleagues showing that CNS neurons can grow into peripheral nerve grafts, but stop when they re-encounter the CNS led to the predominance of the hypothesis that the failure of axons to regenerate is attributed to the presence of inhibitory factors in the CNS microenvironment (David and Aguayo, 1981; Yiu and He, 2006). Indeed, it is now recognized that CNS myelin contains myelin-associated inhibitors (MAIs) including Nogo, myelin-associated glycoprotein (MAG) and oligodendrocyte myelin glycoprotein (OMgp) that collapse axonal growth cones and inhibit growth. The deposition of chondroitin sulfate proteoglycans (CSPGs) by reactive astrocytes also presents a formidable barrier to axon regeneration through sites of injury (Yiu and He, 2006). Targeting these extrinsic inhibitory factors has led to modest improvements in axonal plasticity and functional recovery after CNS injury. Important work spearheaded by Marie Filbin and colleagues revealed a critical role for the intrinsic state of CNS neurons in mediating extrinsic inhibition of axon regeneration. Filbin and colleagues demonstrated that an age-associated decline in neuronal cAMP underlies a gain of sensitivity to myelin-mediated inhibition (Cai et al., 2001). The notion that the neuron-intrinsic growth state can regulate the sensitivity of the injured axon to extrinsic factors is also supported by the enhanced axon regeneration in the spinal cord that can be elicited by a preconditioning lesion of the peripheral nerve processes (Mar et al., 2014a). More recently, it has been shown that stimulating intrinsic growth potential by neuronal knockout of negative regulators of growth, including phosphatase and tensin homolog (PTEN) and suppressor of cytokine signaling 3 (SOCS3), can induce striking long-distance axon regeneration after CNS injury (Liu et al., 2010; Sun et al., 2011). Furthermore, engraftment of neural stem cells into transected rodent spinal cords can result in impressive long-distance growth of grafted cells (Lu et al., 2012). These studies provide proof-of-concept that robust neuron-intrinsic growth potential is able to overcome the inhibitory nature of the injured CNS. Here we will review some novel therapeutic strategies that have been developed based on our expanding knowledge of the molecular basis of intrinsic axon growth and extrinsic outgrowth inhibition (Figure 1). We will also highlight several recent examples of intrinsic regulators that dually affect the neuronal response to the inhibitory CNS environment. These findings have blurred the distinction between an extrinsic or an intrinsic origin of CNS regeneration failure and support the idea that a dynamic interplay between the two determines whether an axon regenerates. A better understanding of this crossroads between extrinsic and intrinsic regulation of axon regeneration will contribute to the conception of therapies to stimulate CNS repair.

## Extrinsic Influences on Neural Repair

### Myelin-associated Inhibitors

The identification of the inhibitory ligands and neuronal receptors that mediate myelin-dependent inhibition of regeneration has facilitated the development of selective antagonists to neutralize the inhibitory effect of myelin proteins. MAIs in CNS myelin bind to either paired immunoglobulin repeat B (PirB; Atwal et al., 2008) or Nogo receptor (NgR), which forms a complex with LINGO-1 (LRR and Ig containing Nogo Receptor interacting protein) and p75NTR (p75 neurotrophin receptor) or TROY (tumor necrosis factor receptor superfamily member 19; Yiu and He, 2006). One strategy to target these interactions has been to generate peptide mimics of ligands to compete for receptor binding. An early example of this is the Nogo extracellular peptide (NEP1–40), a peptide derived from the Nogo-66 NgR-binding region of Nogo to compete with the MAIs for NgR binding (Grandpré et al., 2002). NEP1–40 was shown to abrogate Nogo-dependent neurite outgrowth inhibition and promote corticospinal tract regeneration after dorsal hemisection SCI (Grandpré et al., 2002; Li and Strittmatter, 2003), but a later replication study did not observe significant effects on axon regeneration (Steward et al., 2008). Vaccines (Huang et al., 1999) and monoclonal antibodies (Zörner and Schwab, 2010) have also been developed to neutralize the MAIs, with reported improvements on regeneration and functional recovery. However, deletion of NgR or Nogo, MAG and OMgp in mice has had more variable effects on regeneration and recovery (Zheng et al., 2003, 2005; Lee et al., 2009, 2010; Cafferty et al., 2010). While deletion of Nogo or NgR has negligible to modest effects on axon regeneration after dorsal hemisection SCI, a unilateral pyramidotomy injury to the CST results in an impressive collateral sprouting response of uninjured axons into the denervated side of the spinal cord (Cafferty and Strittmatter, 2006). Furthermore, a soluble NgR decoy receptor, NgR(310)ecto-Fc that neutralizes Nogo, MAG, and OMgp shows promise in promoting axonal sprouting and functional recovery in acute and chronic SCI in rodents (Ji et al., 2005; Li et al., 2005; Wang et al., 2011).

### The Glial Scar

The astrocytic response to CNS injury is characterized by the production of CSPGs, which present a potent barrier to axon regeneration (Yiu and He, 2006). The inhibitory action of CSPGs has been attributed to the abundance of negatively charged glycosaminoglycan (GAG) chains that decorate the protein core, which are thought to act as a poor substrate and electrostatically repel growth cones. Protein tyrosine phosphatase sigma (PTPsigma), leukocyte common antigen related phosphatase (LAR) and NgR have been identified as neuronal receptors that functionally interact with and mediate CSPG-dependent inhibition of neuron growth (Shen et al., 2009; Fisher et al., 2011; Dickendesher et al., 2012). Chondroitinase ABC (chABC), a bacterial enzyme that digests the GAG chains, abrogates CSPG-dependent neurite outgrowth inhibition *in vitro* and improves neurite sprouting and functional recovery after SCI (Bradbury et al., 2002). The efficacy of chABC alone and in combination with other approaches in promoting recovery after SCI has been independently confirmed in multiple laboratories and is a promising approach for future translation (Alilain et al., 2011; García-Alías et al., 2011; Lee et al., 2012). Mechanistically, contrary to the view that CSPGs repel growth cones, recent studies from the Silver group suggest that CSPGs in fact tightly adhere to and confine growth cones (Filous et al., 2014; Lang et al., 2015). After SCI, axons closely associate with NG2, a transmembrane CSPG expressed by a subset of oligodendrocyte progenitor cells. The presence of synaptic markers at these sites of apposition suggests the formation of stable “synapse-like” contacts between neurons and NG2+ cells (Filous et al., 2014). Furthermore, DRG neurons preferentially adhere to CSPG substrates in *in vitro* stripe assays. Silver and colleagues propose a model whereby CSPGs function to capture and arrest regenerating growth cones after injury instead of repelling them (Filous et al., 2014). Perhaps unexpectedly, deletion of NG2 in mice did not enhance axon regeneration after dorsal column crush, but rather resulted in more pronounced axonal dieback away from the injury site. This was attributed to the loss of NG2-mediated capture of axons after injury.

Pharmacological targeting of CSPG receptors is a strategy to relieve CSPG-dependent capture of growth cones that has shown potential in recent studies. Systemic administration of cell-penetrating peptide mimetics of the extracellular and intracellular regions of LAR by subcutaneous injection have been shown to enhance serotonergic axon sprouting and functional recovery after SCI in mice (Fisher et al., 2011). A recent report from the Silver group demonstrates that PTPsigma can be targeted in a similar fashion with a cell–permeable peptide, termed intracellular sigma peptide (ISP), to enhance functional recovery after contusive spinal cord injury in rats (Lang et al., 2015). ISP was designed to bind to the cytoplasmic wedge domain of PTPsigma. Using a CSPG gradient crossing assay, Silver and colleagues provide evidence that PTPsigma-dependent interactions with CSPGs maintain growth cones in a dystrophic state and that treatment with ISP allows for growth cone release. Based on the proposed mechanism that the interaction between PTPsigma and CSPGs acts as a “molecular velcro”, it is surprizing that ISP is sufficient to overcome growth cone immobilization because ISP targets the cytoplasmic domain of PTPsigma, presumably sparing the physical interaction between CSPGs and the receptor. The relative contribution of the receptor-ligand interaction and downstream signaling events to growth cone immobilization remain unclear. Daily subcutaneous injection of ISP following spinal cord contusion in rats resulted in delayed beneficial outcomes, particularly in resumption of bladder function (Lang et al., 2015). While these studies have identified CSPG receptors as potential targets, future work could focus on developing small molecule inhibitors to attain better CNS penetrance and distribution for earlier and more efficient targeting after injury. The idea that CSPGs are critical for growth cone arrest suggests that these approaches may also be efficacious in chronic SCI.

## Intrinsic Influences on Neural Repair

### The Conditioning Lesion

Injury to axons in the PNS is followed by neuronal expression of regeneration-associated genes (RAGs), a response that is quite limited in CNS neurons (Mar et al., 2014a). It has therefore been hypothesized that a failure to initiate a growth program contributes to unsuccessful regeneration in the CNS. The positive effect of a peripheral conditioning lesion on CNS regeneration has been attributed to the engagement of a RAG expression response that stimulates growth (Blesch et al., 2012). One of these RAGs, c-Jun, has been shown to be critical for axon regeneration, as its deletion impairs axon regeneration and results in exacerbated cell death after peripheral nerve injury in mice (Raivich et al., 2004). The conditioning lesion has also recently been shown to enhance the axonal transport of a multitude of signaling and cytoskeleton regulators into the central process (Mar et al., 2014b). Among these include members of the 14-3-3 adaptor proteins, which have important roles in neuroprotection, axon guidance and cell growth (Shimada et al., 2013; Kaplan et al., 2014). Elevation of cAMP is also a critical component of the conditioning lesion effect and injection of cAMP into dorsal root ganglia can mimic the pro-regeneration effect of a conditioning lesion (Qiu et al., 2002). Interestingly, cAMP also regulates the sensitivity of neurons to MAIs and CSPGs (Cai et al., 2001). The Filbin group demonstrated that an age-associated decline of neuronal cAMP correlates with an acquisition of responsiveness to myelin-mediated inhibition of growth and that pharmacological elevation of cAMP renders adult neurons insensitive to myelin and CSPGs (Cai et al., 2001). This reinforces the concept that extrinsic inhibitors rely on the intrinsic sensitivity of the neuron in order to inhibit growth and suggests that targeting neuron-intrinsic signaling can relieve the influence of the inhibitory microenvironment. Supporting a direct link between cAMP and responsiveness to myelin, a recent study shows that enhanced cAMP and protein kinase A (PKA) signaling stimulates the proteolysis of Nogo through activation of the ubiquitin ligase praja2 (Sepe et al., 2014). cAMP is a promising target for development and rolipram, a phosphodiesterase 4 (PDE4) inhibitor that elevates intracellular cAMP, has been shown to enhance regeneration and recovery after SCI in rodents (Lu et al., 2004; Nikulina et al., 2004; Pearse et al., 2004; Kadoya et al., 2009; Costa et al., 2013).

### MAI Signaling

The actin-regulating small GTPase RhoA and downstream effector Rho kinase (ROCK) are extensively studied mediators of neurite outgrowth inhibition in the CNS. MAIs have been shown to increase the levels of active GTP-bound RhoA (Dubreuil et al., 2003) and one study suggests that the dual Rac and Rho guanine exchange factor (GEF), Kalirin9, functionally interacts with p75 in complex with NgR1 to stimulate RhoA activation in response to receptor activation (Harrington et al., 2008). Nogo also stimulates the phosphorylation and membrane translocation of ROCK (Alabed et al., 2006). Inhibition of ROCK stimulates neurite outgrowth on myelin and infusion of the small molecule ROCK inhibitors Y-27632 or fasudil into the injured rat spinal cord improves locomotor recovery (Fournier et al., 2003; Sung et al., 2003). However, while ROCK inhibition enhances neurite outgrowth on myelin, a persistent myelin-induced reduction of growth relative to enhanced baseline growth on permissive substrates is often reported (Fournier et al., 2003; Alabed et al., 2006; Ahmed et al., 2009), suggesting that ROCK inhibition fails to fully de-sensitize neurons to myelin-dependent growth inhibition. It is therefore difficult to attribute the positive effects of ROCK inhibition in SCI to a reduction in the sensitivity of the axons to myelin or simply to an intrinsic stimulation of outgrowth. Our group has identified a Nogo-induced interaction between RhoA and collapsin response mediator protein 4 (CRMP4) as a functional signaling event that can be targeted with a competitive peptide antagonist called C4RIP to block myelin-induced neurite outgrowth inhibition (Alabed et al., 2007). Interestingly, C4RIP does not enhance baseline neurite outgrowth (Alabed et al., 2007), suggesting the specific involvement of a CRMP4-RhoA complex engaged by MAIs. RhoA can be specifically inhibited with the *Clostridium botulinum* exoenzyme C3 transferase, which ADP-ribosylates and inactivates RhoA. Treatment of animals with C3 improves locomotor recovery in contusion and dorsal hemisection injury models (Boato et al., 2010). C3 has a dual effect of enhancing neurite outgrowth in the presence of myelin (Dergham et al., 2002) and promoting cell survival after SCI (Dubreuil et al., 2003). Interestingly, C3 has also been shown to stimulate neurite outgrowth and induce Erk and Akt phosphorylation independent of its ability to inhibit RhoA, suggesting that its mechanism-of-action has yet to be fully elucidated (Auer et al., 2012). Detrimental effects of C3 (Fournier et al., 2003; Sung et al., 2003) observed in other studies are potentially due to endotoxin contamination and lack of a cell-penetrating peptide (McKerracher and Higuchi, 2006). The positive outcomes with C3 have translated into a phase I/II clinical trial with Cethrin (BA-210), a cell-permeable form of C3 that is delivered locally over the dura mater in a fibrin sealant during spinal surgery. Results from the Cethrin trial have shown promising improvements of locomotor function compared to historical statistics (McKerracher and Anderson, 2013).

### Growth Stimulation

Recent studies have provided a strong rationale for targeting intracellular growth regulators as a means to both drive neuron growth and relieve sensitivity to extrinsic inhibition of growth. Initial studies have explored strategies to stimulate intrinsic growth potential by genetically manipulating the expression of master regulators of cellular growth that are considered classical tumor suppressors or oncogenes. PTEN is a tumor suppressor that antagonizes the PI3K-Akt-mTOR pathway (Song et al., 2012). The He lab reported an age-associated down-regulation of mTOR activity in adult cortical neurons and showed that conditional knockout of PTEN and consequent increase in mTOR activity in mouse cortex promotes regeneration of corticospinal axons after dorsal hemisection injury (Liu et al., 2010). Another study showed that combined deletion of PTEN and SOCS3, a negative regulator of the JAK/STAT pathway, synergize to promote axon regeneration in injured optic nerve (Sun et al., 2011). These studies were noted for extraordinarily extensive long-distance axon regeneration that had not been observed with any prior intervention. These positive outcomes have resulted in a greater focus on manipulation of intrinsic regulation of neuron growth as an approach for drug development. Chronic targeting of tumor suppressors poses a concern for oncogenesis, but an acute regimen may be an effective strategy to initiate a regenerative response after injury. One study found that systemic administration of the PTEN inhibitor bisperoxovanadium (bpVpic) twice daily for 1 week improved forelimb motor function and was neuroprotective (Walker et al., 2012).

A complementary strategy is to enhance the activity or expression of growth-promoting molecules. A recent study showed that expression of a point-mutated kinase activated version of the B-Raf oncogene (V600E B-Raf) results in robust regeneration of retinal axons after optic nerve injury and regeneration of dorsal root ganglion neurons into the dorsal root entry zone after crush injury (O’Donovan et al., 2014). Further enhancement of regeneration was observed with combined knockout of PTEN. How these manipulations influence neuronal responses to extrinsic inhibition are poorly understood. It was recently shown that codeletion of Nogo and PTEN does not cause more corticospinal axon sprouting after unilateral pyramidotomy, but results in improved long-distance axon regeneration after dorsal hemisection, compared to PTEN deletion alone (Geoffroy et al., 2015). The lack of a combinatorial effect of PTEN and Nogo deletion on sprouting suggests that perhaps PTEN deletion affords maximal de-sensitization to Nogo. One study provides evidence that PTEN knockout neurons are less sensitive to MAG-dependent neurite outgrowth inhibition (Perdigoto et al., 2011), while another study has shown that Erk, a downstream effector of Raf, can also relieve MAG-dependent inhibition (Gao et al., 2003). It remains unclear whether these pathways are directly involved in MAI signaling or whether they act in parallel to alter MAI signal-transduction efficiency. Interestingly, a recent study shows that myelin and CSPGs stimulate the expression of pro-growth immediate early genes through serum response factor (SRF) and Erk activation. This may function as a protective response because expression of constitutively active SRF was shown to overcome myelin and CSPG—dependent neurite outgrowth inhibition (Stern and Knoll, 2014).

### Neural Stem Cells

The ability of implanted neural stem cells to survive and functionally integrate into injured host spinal cord in rodents also suggests that the inhibitory nature of the injured CNS can be overcome by neurons with vigorous growth capacity (Lu et al., 2012, 2014). In studies from the Tuszynski group, implanted neural stem cells were shown to extend long axons throughout the grey and white matter of transected host spinal cords, establishing an electrophysiological bridge across the injury. A recently published study confirmed the ability of grafted neural stem cells to integrate into host spinal cords, but also noted the presence of ectopic colonies of donor cells throughout the spinal cord and brain stem in half of the animals (Steward et al., 2014). This highlights the caution that must be exercised in the development of neural stem cell therapies, as implanted cells can give rise to tumors and exuberant synaptic connections could result in unfavorable behavioral and sensory side effects including neuropathic pain (Hofstetter et al., 2005). Nonetheless, these studies also serve as proof-of-principle that neurons with high growth capacity are capable of extensive growth in the injured CNS despite the presence of inhibitory factors.

## Conclusion

Both the extrinsic and intrinsic aspects of the injury response together forge the basis for regeneration failure. Peripheral conditioning lesions combined with chABC and NgR(310)ecto have been shown to provide superior benefit to single interventions, supporting the idea that simultaneously stimulating neuron-intrinsic growth potential and neutralizing extrinsic inhibition can provide maximal efficacy in promoting neural repair (Wang et al., 2012). SCI therapies that take both of these contributors into consideration can come in the form of drug combinations or single multi-action drugs. The use of multi-action drugs is an attractive strategy to simultaneously manipulate several pathophysiological features of the injury response. For example, work from the Bradke group has shown that local administration of low dose taxol, a potent microtubule-stabilizing drug, exerts manifold beneficial effects by reducing CSPG production and fibrosis, inhibiting meningeal fibroblast migration and stimulating axon extension after SCI (Hellal et al., 2011). A recent report from the Bradke group shows that systemic administration of another microtubule stabilizer, epothilone B, similarly inhibits scarring and promotes axon regeneration (Ruschel et al., 2015). Both drugs are approved chemotherapeutics, however epothilone B, but not taxol, is CNS-penetrant. CNS-penetrant multi-action pharmaceutical agents that can be given systemically hold the most promise for translation into practical and effective treatments for SCI. The repurposing of approved drugs for indication in SCI is an attractive approach to achieve accelerated approval for this serious unmet need. However, the identification of new drugs that have multiple activities, including modulation of astrogliosis, neuroprotection and neurite outgrowth induction, may offer superior efficacy in stimulating CNS repair, but will also pose challenges for safety and tolerability.

## Author Contributions

AK, SOT and AEF wrote the manuscript.

## Conflict of Interest Statement

The authors declare that the research was conducted in the absence of any commercial or financial relationships that could be construed as a potential conflict of interest.
